# Contactless radar-based breathing monitoring of premature infants in the neonatal intensive care unit

**DOI:** 10.1038/s41598-022-08836-3

**Published:** 2022-03-25

**Authors:** Gabriel Beltrão, Regine Stutz, Franziska Hornberger, Wallace A. Martins, Dimitri Tatarinov, Mohammad Alaee-Kerahroodi, Ulrike Lindner, Lilly Stock, Elisabeth Kaiser, Sybelle Goedicke-Fritz, Udo Schroeder, Bhavani Shankar M. R., Michael Zemlin

**Affiliations:** 1 SnT - Interdisciplinary Centre for Security, Reliability and Trust, University of Luxembourg, Luxembourg, Luxembourg; 2 Department of General Pediatrics and Neonatology, Saarland University Medical School, Homburg, Germany; 3 IEE S/A, Bissen, Luxembourg

**Keywords:** Neonatology, Paediatric research, Preterm birth, Biomedical engineering, Electrical and electronic engineering

## Abstract

Vital sign monitoring systems are essential in the care of hospitalized neonates. Due to the immaturity of their organs and immune system, premature infants require continuous monitoring of their vital parameters and sensors need to be directly attached to their fragile skin. Besides mobility restrictions and stress, these sensors often cause skin irritation and may lead to pressure necrosis. In this work, we show that a contactless radar-based approach is viable for breathing monitoring in the Neonatal intensive care unit (NICU). For the first time, different scenarios common to the NICU daily routine are investigated, and the challenges of monitoring in a real clinical setup are addressed through different contributions in the signal processing framework. Rather than just discarding measurements under strong interference, we present a novel random body movement mitigation technique based on the time-frequency decomposition of the recovered signal. In addition, we propose a simple and accurate frequency estimator which explores the harmonic structure of the breathing signal. As a result, the proposed radar-based solution is able to provide reliable breathing frequency estimation, which is close to the reference cabled device values most of the time. Our findings shed light on the strengths and limitations of this technology and lay the foundation for future studies toward a completely contactless solution for vital signs monitoring.

## Introduction

Nearly 15 million infants are born annually before the 37th week of pregnancy, meaning that about 10% of all births worldwide are premature^1^. Due to their immature organ systems and associated functions, as well as their immune system, these infants are at a higher risk of infections, chronic diseases and respiratory problems. The immaturity of breathing regulation and lungs often lead to apnea-bradycardia and respiratory distress syndromes. This is commonly followed by bronchopulmonary dysplasia in 27% of the infants born at less than 30 weeks of gestation^2–5^. Consequently, further development of these premature infants has to continue *ex-utero*, and they usually have to spend several weeks at a Neonatal Intensive Care Unit (NICU).

During this period, continuous monitoring of their underdeveloped organs is necessary. Often, newborns are dependent on parenteral nutrition, respiratory support, and invasive diagnostic interventions which, albeit being essential for survival, may cause stress to the child. Basic vital parameters such as respiration, heart rate and oxygen saturation also need to be monitored. To this end, several sensors are directly attached to their fragile skin and connected to the monitoring systems through cables. Besides mobility restrictions, these sensors often cause skin irritation and may eventually lead to pressure necrosis^6–11^.

In order to promote the development of premature babies, a number of efforts have been made toward non-invasive monitoring and diagnostic solutions. The use of sensors that can monitor a variety of vital signs without a cable connection, but bonded to the skin, is being investigated in^12,13^. Current studies are also investigating the potential of different non-contact techniques for non-invasive diagnostics in children. Efforts are underway to detect pathological changes in body excretions by analyzing volatile organic compounds^14,15^. There are approaches using optical methods to monitor pulse rate and oxygen saturation without direct skin contact and cable connection, based on e.g. dynamic light scattering^16^, video^17^ or photoplethysmography^18,19^. Of high relevance for preterm infants is also the diagnosis of respiratory pathologies and classification regarding periodic breathing and apneas^20,21^. This task is addressed using different non-contact techniques, which require redundant measurements of various vital signs, e.g. respiration motion, heart rate, oxygen saturation or nasal breathing^22–24^.

The contactless monitoring of the cardiorespiratory activity neither confines nor inhibits the patient, reduces hygiene risks and does not cause any discomfort, irritation or skin damage^25,26^. In this context, radars have already been proven to be a promising technology^27–29^, being intrinsically low-power, low-cost and privacy preserving. Unlike camera-based systems^30,31^, radar signals can penetrate through different materials (such as plexiglass, clothing, mattresses and blankets), and are not affected by skin pigmentation or ambient light levels. However, due to the reduced transmitted power, these signals can be easily buried in the background noise, or masked by stronger external interference, including body movements from the monitored patient^32^. This interference is a major challenge for accurate estimation in contactless solutions as well as for cabled devices. Specific signal processing techniques are thus needed in order to ensure reliable and robust measurements.

Recent works^33,34^ have demonstrated that an ultrawideband radar can provide reliable breathing rate estimates for neonates under specific conditions. However, these investigations were limited to a single scenario, where the neonates were lying over an open-air crib, always in supine position. In addition, radar performance was evaluated only during minimal movements of the monitored patients. In this article, we take one step further by using a simpler continuous-wave (CW) radar device, and investigating premature infants under different scenarios common to the NICU routine, irrespective of the amount of movement or external interference. The specificity of the monitored patients in a real clinical setup creates several challenges which were addressed through different contributions in the proposed signal processing framework. Particularly, rather than just discarding measurements under strong interference^33–37^, we present a novel random body movement mitigation technique based on the time-frequency decomposition of the recovered signal. Additionally, we propose a simple and accurate frequency estimator, which explores the harmonic structure of the breathing signal.

### Problem formulation

**Figure 1:**
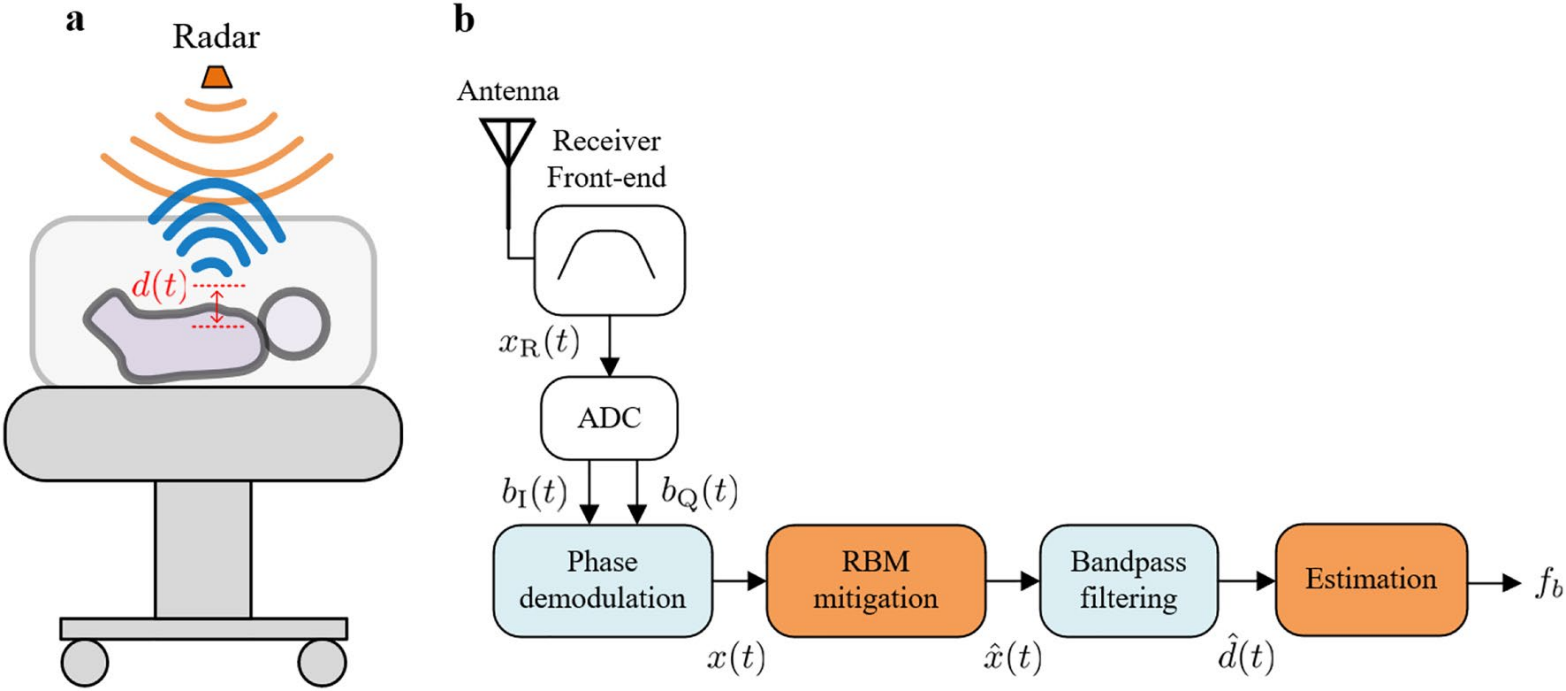
Continuous-wave radar for breathing monitoring in the NICU. (**a**) Basic operational principle. (**b**) Block diagram of the signal processing chain. Before estimation, the received signals from the analog-to-digital converter (ADC) are phase demodulated and further processed in the random body movements (RBM) mitigation unit.

The activity of the cardiovascular and respiratory systems causes some physical and physiological effects on the human body. The chest wall moves during the inspiration/expiration cycle as a result of the diaphragm and intercostal muscle movements. This small and periodic displacement can be detected by radar, allowing accurate estimation of the breathing rates under certain conditions. Figure 1a illustrates the basic operational principle of a CW radar. The transmitted signal propagates through the free space and reaches every object in the radar’s field-of-view, being reflected back with additional phase information regarding each object’s position. The received signal can thus be modeled as a scaled and time-shifted version of the transmitted signal, in which the phase variation over time contains valuable information regarding the scene movement. This time-varying phase $\theta (t)$ can usually be recovered as

1 $$\begin{aligned} \theta (t) = \frac{4 \pi d(t)}{\lambda }, \end{aligned}$$

where $\lambda$ is the radar operating wavelength, and *d*(*t*) represents the displacement signal which, ideally, would correspond only to the chest wall motion due to the breathing mechanism. As seen by the radar, this movement is mainly originated by the reflected points over the chest moving surface, but it may additionally include residual motion from the belly, sides and the back, depending on the patient relative position. In healthy adults, standard amplitudes for this motion range between 4 mm to 12 mm^38^, with breathing rates varying from 5 to 25 breaths per minute (bpm)^39^. For premature infants, these amplitudes can be smaller than 1 mm, while average breathing rates can normally reach 60 bpm^40^, and go up to 80 bpm under specific conditions^41^.

Perfect recovery of the chest wall motion *d*(*t*) would allow precise estimation of the breathing frequency $f_b$ by simple analysis of the movement periodicity. However, in a real clinical setup, besides unavoidable hardware imperfections, the received radar signal is usually mixed with additional reflections from the external environment, arising not only from different body movements of the monitored patient, but also from every moving object in the scene. These interfering signals are usually much stronger than those induced by the chest wall millimeter displacement, and this makes accurate recovery and subsequent estimation of the breathing frequency a challenging task. In addition, when considering premature infants, the reduced amplitudes of the chest wall motion, and the wider range of possible breathing rates pose an additional signal processing challenge in relation to previously reported research with adults.

**Figure 2:**
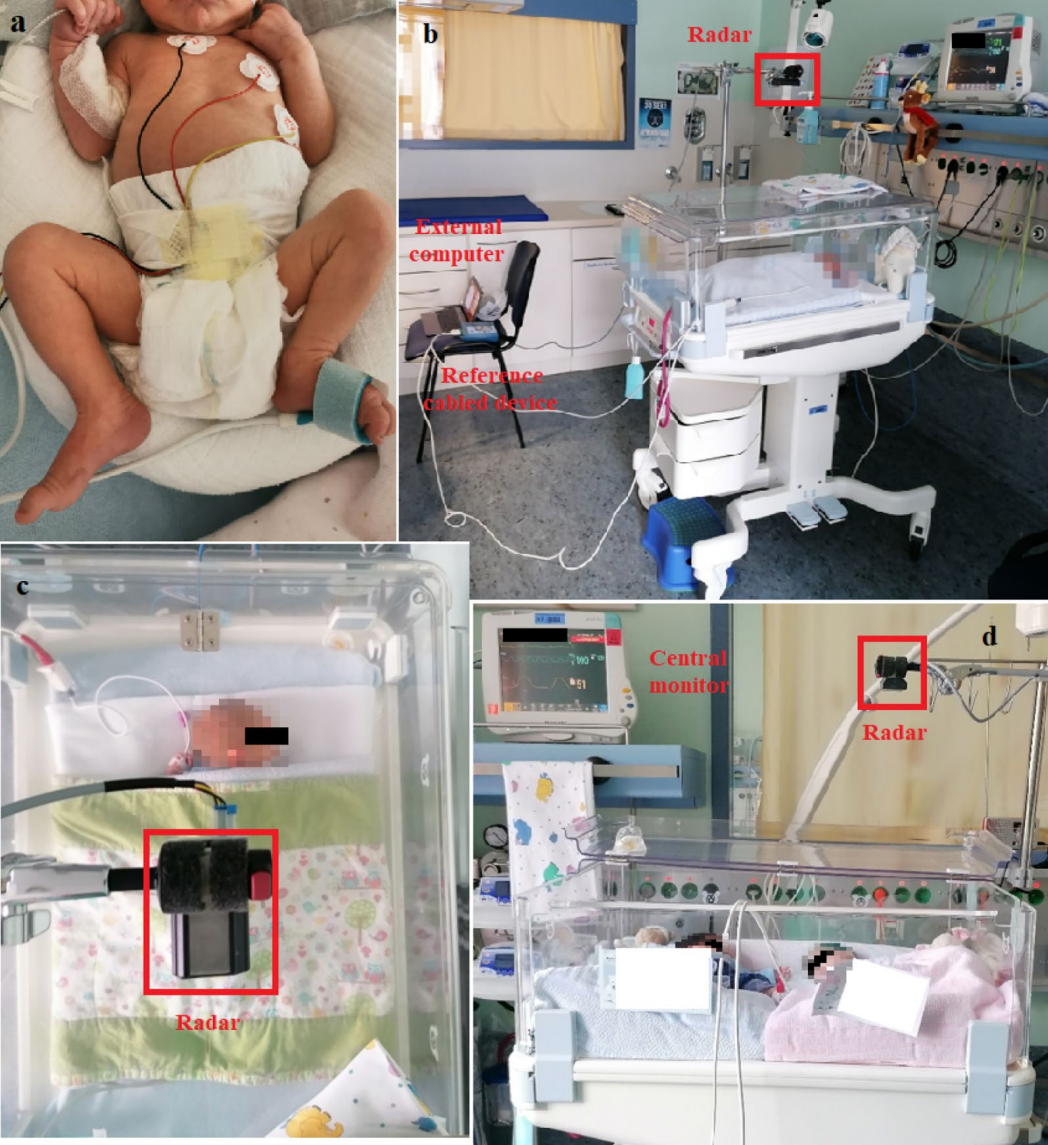
Clinical setup. (**a**) Conventional monitoring of premature neonate: connection by cables to the central monitoring unit (heart rate, oxygen saturation, respiration), and an additional peripheral venous catheter. (**b**) NICU room view. Both radar and the reference cabled device were controlled from the external computer. (**c**) Close top view. The radar was attached to a low-vibration tripod, 45 cm away from the infant. (**d**) Close side view with twins sharing the same bed.

### Clinical setup and protocol

The study was performed in the Department of Pediatrics, at the Saarland University Medical Center (Homburg, Germany). Figure 2a shows a premature neonate being monitored with the conventional method. Besides the sensors attached to the chest and abdomen, and connected by cables to the central monitoring unit (for oxygen saturation, heart rate, and respiration), an additional peripheral venous catheter and a gastric tube are also necessary in this stage. The clinical setup, including the neonatal cot, the radar device, and the reference monitoring system is shown in Fig. 2b–d. The radar is certified for operation in the 24-GHz ISM (industrial, scientific and medical) band, and it was installed outside the cot, attached to a low-vibration tripod. The relative distance to the monitored infant was around 45 cm to 50 cm. Due to the radar’s inherent capabilities, no modification to the cot structure was necessary, and the plastic cover could remain closed during the measurements. In Fig. 2d, twins are sharing the same bed, with only one being monitored with the contactless method. Cobedding of twins is a common procedure in the NICU, with several studies reporting physiological benefits to the infants^42,43^.

A total of 12 premature infants were included in the study. The Supplementary Table 1 shows a summary of the patient’s information. They were selected on the basis of medical opinion, and taking into account the medical safety of participating in the study. For each infant, the measurements were carried out in three different days, at noon (after feeding), over a period of 25 minutes each. Their natural position was not changed during each measurement. Besides the supine (with the chest facing the radar), prone (with the back facing the radar) and side positions, we have also investigated cobedding cases with only one infant being monitored using the contactless method. The idea was to investigate the different effects when collecting radar data from the chest/abdomen and back. Additionally, if monitoring twins is possible, and what would be a safe distance (in terms of radar interference) between them. The basic principle that guided the data collection protocol was to ensure seamless operation at the NICU. A detailed description of the patient’s protocols is shown in Supplementary Tables 2a–d, including all interventions and additional transients manually annotated during the measurements.

### Signal processing background

Figure 1b shows the basic block diagram of the signal processing chain. The initial signal processing step for CW systems is commonly known as phase demodulation. It is essentially the process where the received in-phase and quadrature (I and Q) signals from the radar’s analog-to-digital converter (ADC) are combined with the aim to recover the displacement signal *d*(*t*). Among several methods, the two most used are the arctangent demodulation (AD)^44^ and the complex signal demodulation (CSD)^45^. While the AD enables precise recovery of the chest wall motion, it is highly sensitive to hardware calibration, and to the presence of DC offsets, noise, and external interference. The CSD is more robust to these effects, but it relies on small displacements for recovering an approximation of the breathing motion (please refer to the Methods).

**Figure 3:**
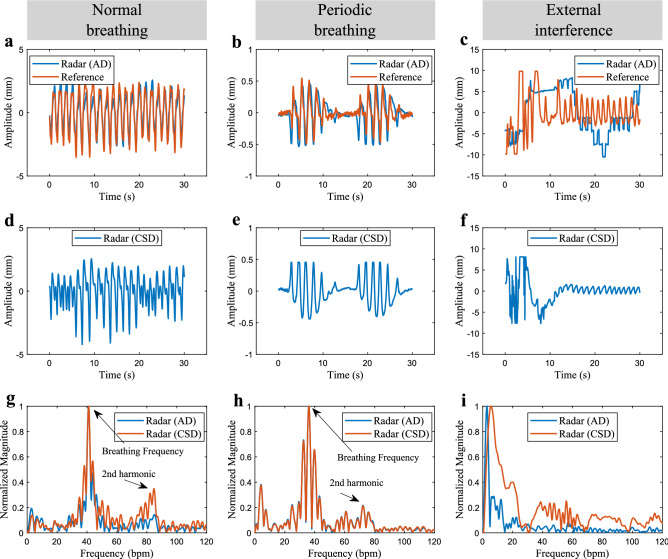
Recovered chest wall motion with different breathing patterns. (**a**) Normal breathing pattern, under good conditions (no interference). (**b**) Periodic (Cheyne-Stokes) breathing pattern. (**c**) Normal breathing pattern corrupted by external interference and ADC saturation. (**d–f**) Approximation using the CSD. (**g–i**) Spectrum comparing AD and CSD.

Figure 3 shows examples of the recovered breathing motion from radar data, in comparison to the actual (reference) displacement acquired from the cabled device. Initially, to precisely reconstruct the chest wall displacement, we selected “clean” segments of data (no external interference), and the AD was used in both cases. While Fig. 3a depicts a normal breathing pattern obtained at supine position, Fig. 3b shows an occurrence of the Cheyne-Stokes (periodic) breathing pattern^46^, with the infant at prone position. This special form of breathing is physiologically found in neonates, and is defined by a cyclic variation between hyperpnea and hypopnea^47–49^, i.e. repetitive short cycles of pauses and breaths. Despite small differences between the recovered radar signals and the reference device, the periodic breathing movement can still be clearly identified in both cases. The small amplitudes of the chest wall motion can also be visualized, with displacements around 2 mm in supine position and 0.5 mm in prone position. These amplitudes are well below typical values for adults reported in previous research^50–52^.

The approximated breathing motion, obtained with the CSD, is shown in Fig. 3d,e. Despite noticeable differences when compared to the AD, Fig. 3g,h show that both techniques yield signals with the same fundamental frequency, corresponding to the average breathing frequency. Given the small displacements we aim to detect, and the challenging conditions of this real clinical environment, the CSD was adopted in our solution for long-term monitoring. The harmonic structure of the breathing signal can also be visualized, with the second harmonic being clearly distinguishable. This harmonic structure can be used for improving estimation, as we will show latter. However, as depicted in Fig. 3c,f,i, under external interference and eventual ADC saturation, both demodulation methods fail to reconstruct the chest wall motion. The spectrum becomes dominated by the interference components, which will then prevent accurate breathing frequency estimation. Therefore, additional processing is necessary in order to attenuate these effects.

### Random body movement mitigation

Most research on contactless vital sign monitoring with radar sensors focus on a single-person setup under ideal motionless conditions^53^. In practical monitoring situations, the subject may often move body parts like hands, legs or torso, and even the entire body. These unwanted but unavoidable movements are usually called random body movements (RBM). The amplitude of their reflected signals is often much stronger than the millimeter-scale breathing motion, which will potentially be masked by this interference. Since spontaneous RBM are inevitable, solving this problem is fundamental to reliable vital sign detection in practical applications.

Several methods for RBM mitigation were already proposed^54^, and even though specific types of movements could be effectively cancelled out, they usually require more complex systems. Most solutions rely on additional or duplicated hardware, thus suffering from practical limitations such as misalignment, synchronization, and cost^45,55,56^. Another direction of research basically tries to identify segments of vital sign data with RBM, and simply discard these corrupted segments before estimation^33–37^. However, depending on the processing window duration, even very short RBM will affect several seconds of good signal. Therefore, rather than simply discarding segments of data, an approach which allows useful exploitation of these episodes with moderate RBM is desired. Recent work has begun to address RBM using a single sensor and within more challenging scenarios^32^. Nonetheless, experimental validation is still performed under controlled situations, with RBM being emulated through predefined behavior, which results in limited interference over the desired signal.

First, let us assume that RBM are sparse, i.e. they are not frequent and, when they occur, their duration is small in relation to the observed time window. This contrasts with the constant and periodic nature of the breathing movement. Additionally, their amplitudes are usually much stronger than the standard breathing signal. These specific time and frequency features will be present in the spectrogram of the recovered signal, which can be analyzed toward identifying and possibly removing this interference. For addressing this, we will use the nonnegative matrix factorization (NMF)^57,58^, a matrix decomposition technique usually employed for extracting features from a set of nonnegative data. If *x*(*t*) (Fig. 1b) is the recovered signal containing the chest wall motion and eventual RBM interference, its magnitude spectrogram $|{{\varvec{X}}}|$ can be obtained through the Short-Time Fourier Transform (STFT) of *x*(*t*). The NMF will then decompose $|{{\varvec{X}}}|$ as

2 $$\begin{aligned} |{{\varvec{X}}}| \approx {{\varvec{W}}}{{\varvec{H}}}= \sum _{i=1}^{K} {{\varvec{w}}}_i {{\varvec{h}}}^{\mathrm{T}}_i, \end{aligned}$$

where the matrices ${{\varvec{H}}}$ and ${{\varvec{W}}}$ contain, respectively, the associated time and frequency basis components of $|{{\varvec{X}}}|$, with *K* being a predefined number of basis. In other words, ${{\varvec{W}}}$ can be seen as the set of frequency templates of $|{{\varvec{X}}}|$, while ${{\varvec{H}}}$ contains the timing information related to the activation of these templates. If we look into the time activation matrix ${{\varvec{H}}}$, the basis components with sparse behavior and higher amplitudes will often indicate the epochs when the RBM interference is present. Despite the unpredictable frequency spectrum, which will eventually overlap with breathing frequencies, the RBM distinct time behavior can be captured by the NMF time activation bases ${{\varvec{H}}}$, whereas the corresponding bases in ${{\varvec{W}}}$ will retain its frequency content. This allows additional flexibility for filtering the RBM interference when compared to standard spectral analysis methods. We can thus reconstruct the filtered spectrogram $\hat{|{{\varvec{X}}}|}$, by simply adding back all the ${{\varvec{w}}}_i {{\varvec{h}}}^{\mathrm{T}}_i$ matrices, except for the ones containing the interfering components.

**Figure 4:**
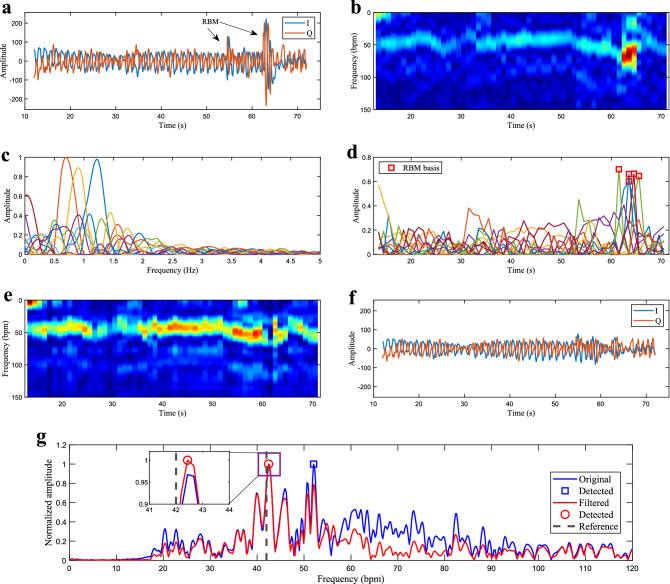
NMF for random body motion mitigation. (**a**) I and Q samples of the displacement signal *x*(*t*), corrupted by RBM. (**b**) Normalized spectrogram $|{{\varvec{X}}}|$. The RBM interference clearly dominates the spectrum and would jeopardize estimation. (**c**) NMF decomposition into the frequency basis components in ${{\varvec{W}}}$. (**d**) NMF decomposition into the time basis components in ${{\varvec{H}}}$. (**e**) RBM filtered spectrogram $\hat{|{{\varvec{X}}}|}$. (**f**) I and Q samples of the RBM filtered time signal ${\hat{x}}(t)$. (**g**) Bandpass spectrum of the original and RBM filtered signals.

Figure 4a shows a 60-s processing window for illustrative purposes, where the recovered signal *x*(*t*) (after CSD) is corrupted by segments of RBM, with its normalized spectrogram $|{{\varvec{X}}}|$ in Fig. 4b. In the case of the CSD, the spectrogram is calculated based on the complex samples of the recovered signal *x*(*t*), and therefore considers both I and Q channels simultaneously. The NMF decomposition into $K=11$ frequency (${{\varvec{W}}}$) and time basis (${{\varvec{H}}}$) components is depicted in Fig. 4c,d, where each color represents a pair of basis components, with the frequency content in Fig. 4c, and the corresponding time activation in Fig. 4d. It can be seen that (please refer to the green and blue bases for instance), due to its random nature, the RBM interference has frequency components spread over the entire spectrum, overlapping with the breathing frequency region. While a variety of frequencies can be visualized in ${{\varvec{W}}}$, the sparse and strong bases corresponding to RBM can be clearly identified in ${{\varvec{H}}}$ (please refer to the Methods). Removing the selected bases allows the reconstruction of the filtered spectrogram in Fig. 4e, where the breathing frequency variation over time (around 45 bpm) is now evident. After the inverse STFT, the RBM filtered time signal is depicted in Fig. 4f. Finally, Fig. 4g shows the bandpass spectrum of both the original and the RBM filtered signals. The corresponding detected values are highlighted respectively with the blue and red markers. The dashed black line shows the reference value for the average breathing frequency associated with this processing window. Because of the strong RBM interference, the maximum value of the original spectrum would indicate an erroneous breathing frequency of 52.1 bpm, very distant from the true value of 42 bpm. After RBM filtering with the NMF, the modified spectrum indicates a closer value of 42.4 bpm, where the estimation error would be only 0.4 bpm.

### Breathing rate estimation

Different models have already been proposed for representing the back-and-forth breathing movement *d*(*t*), from simple sinusoidal approximations^59,60^, to more complicated patterns as described in^38,61^. The breathing movement is a complex phenomenon which involves different patterns of motion, not only from the chest wall surface, but also from the belly, shoulders and back^62,63^. Therefore, it is difficult to identify time-domain models that fully characterize it in a robust way, for every subject and monitoring scenario. However, due to the inherent periodic nature of breathing, any function representing this movement can eventually be decomposed into Fourier terms, containing the fundamental frequency and harmonics that correspond to the breathing rates we aim to estimate. Hence, the displacement signal can be modeled as a sum of harmonically related complex sinusoids, having frequencies that are integer multiples of the fundamental breathing frequency. To better exploit this harmonic structure, in this section we propose a simple and accurate Nonlinear Least Squares (NLS) estimator^64^, which is asymptotically efficient for large processing windows, even in colored noise scenarios^65^.

Initially, for removing any residual DC values, and possible high frequency noise components, the RBM-filtered displacement signal ${\hat{x}}(t)$ is further filtered using a bandpass Kaiser window ($\beta =6.5$), from 0.3 Hz to 3 Hz ($18-180$ bpm). This corresponds to the physiological range of breathing frequencies, also including possible harmonics. The bandpass filtered signal ${\hat{d}}(t)$ will ideally be an accurate approximation of the true chest wall motion *d*(*t*) (Fig. 1), and can finally be used for breathing frequency estimation.

Before estimation, for improving the signal-to-noise ratio (SNR)^66–68^, we calculate the autocorrelation function *r*(*t*) of the bandpass filtered signal. The estimation is first performed in time domain, directly over the autocorrelated signal. An initial (coarse) estimation is obtained through a peak detection algorithm, where the time distance between peaks provides an estimation of the time between each breath. Eventually, detected peaks can be excluded if the distance to its neighbors correspond to a frequency outside the expected physiological range. The initial breathing frequency is thus calculated as the inverse of the time between selected peaks, averaged over the entire processing window. This initial estimation will be used to simplify the NLS algorithm.

The following step is to calculate the NLS frequency estimates ${\hat{\omega }}$, which are obtained by maximizing the similarity between ${\hat{d}}(t)$ and the displacement signal model in (12). Under certain conditions (please refer to the Methods), the solution to this problem (the resulting cost function in (17)) can be efficiently implemented using a Fast Fourier Transform (FFT) and a linear grid search algorithm^69^, where the estimator reduces to a summation of the breathing harmonics over the power spectral density of ${\hat{d}}(t)$. The initial time domain estimation is used for limiting the search range, thus avoiding stronger low-frequency components which may still be present in real data. This strategy also reduces the computational effort to perform the grid search.

## Experimental results

The initial measurements were used for setting up the ideal distance between radar and the monitored infant (Supplementary Fig. 1). From a total of 30 measurements at the adjusted position (around 45 cm), 3 were excluded due to recording issues with the devices. The remaining sequences were processed using sliding windows of 30 s, with 28 s of overlap, leading to updated breathing rate estimates every 2 s. This resulted in approximately 20,250 estimates, from 675 minutes of analyzed data. The measurements proceeded irrespective of the amount of movement or external interference. The sequences include moments with hiccups, yawn, cry, grunt, periodic breathing, and also additional movements from the NICU room, e.g. medical interventions and visitors (Supplementary File 3). All the data processing was performed using Matlab^70^.

**Figure 5:**
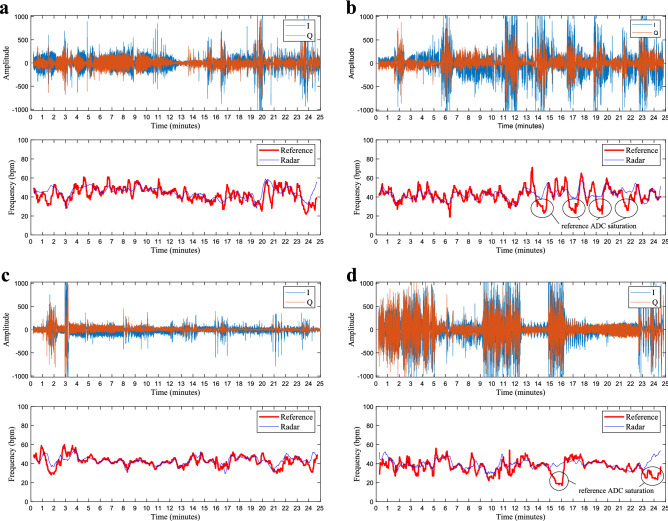
Radar input data and estimated breathing rate. All sequences were processed with the complete proposed solution (NLS+NMF), and each one represents a different monitoring scenario. (**a**) Single infant in supine position (chest facing the radar). (**b**) Single infant in prone position (back facing the radar). (**c**) Twins in the same bed, spaced by 20 cm, with the monitored infant in prone position. (**d**) Highly interfered sequence.

Figure 5 shows examples of I and Q samples from the radar’s ADC, the estimated breathing frequency values, and the reference values from the cabled device. In Fig. 5a, the monitored infant was sleeping calmly, in supine position, and a nurse was present in the NICU room during the entire measurement, 2.5 meters away from the baby. These conditions resulted in good radar measurements with low level of interference. On the other hand, Fig. 5b shows one example where the infant was lying in prone position. In this case, several segments of strong interference can be visualized in the I/Q input data. Besides the infant moving freely and grunting, a nurse was also present during this measurement, from minute 9 and onwards. Figure 5c corresponds to one of the twin cases. A direct intervention over the monitored infant was registered around the third minute, for fastening the oxygen saturation sensor. The second twin was sleeping calmly during the entire measurement. The distance between them was around 20 cm. Figure 5d shows another example of a highly interfered sequence, where the monitored infant was constantly moving and accompanied by the mother, just 1 meter away. In this case, long segments of strong interference (and eventual ADC saturation) can be visualized.

Importantly, when the babies were moving, a high level of saturation was also observed in the reference device input data. During saturation, the ADC clips the input signal at the maximum allowed magnitude, generating segments of constant amplitude that increase the low-frequency content of the current processing window. This limitation results in a rapid decrease in the reference breathing frequency values (valleys), followed by an immediate recovery to the correct values just after saturation ends (these valleys are highlighted in Fig. 5b,d). This anomalous behavior is not related to any physiological pattern, and indicates that the reference device is not reliable in these moments. Consequently, for calculating the performance metrics, we have only considered the processing windows corresponding to non-saturated data segments of the reference device. This resulted in 18 valid measurements and approximately 4,964 valid breathing rate estimates. The measurements from the side position resulted in low SNR and unreliable estimates that were also discarded. On the other hand, radar saturation is being considered inherent to the measurement setup. Therefore, radar data segments with saturated I and/or Q samples are being considered valid and processed.

**Figure 6:**
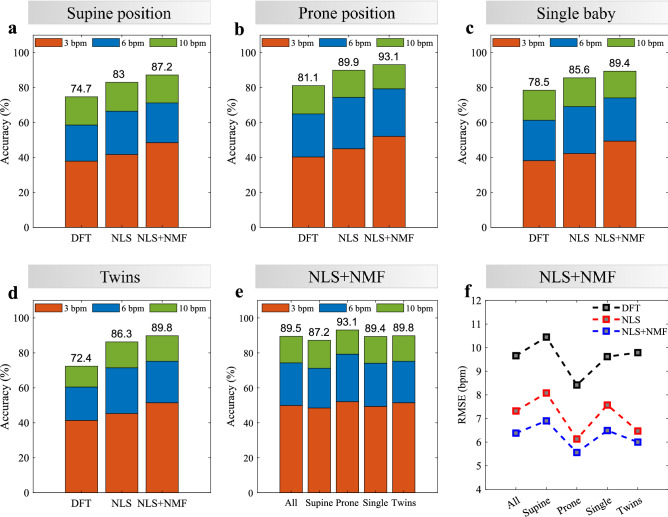
Summary of results, considering all processed sequences for each scenario, and comparing different techniques: standard DFT estimation (DFT), the proposed NLS estimation (NLS), and the complete proposed solution, with RBM mitigation (NLS+NMF). Average accuracy for different techniques. (**a**) Supine position. (**b**) Prone position. (**c**) Single infant in the cot. (**d**) Twins sharing the cot. (**e**) Average accuracy for different scenarios, considering the complete proposed solution (NLS+NMF). (**f**) Average RMSE for different scenarios and different techniques.

A summary of the obtained results is presented in Fig. 6. Figure 6a–d shows the final average (3, 6 and 10 bpm) accuracy, in each of the investigated scenarios, namely: (a) supine position (11 measurements), (b) prone position (7 measurements), (c) single infant in the warming bed (14 measurements) and (d) twins sharing the same bed (4 measurements). These scenarios are not exclusive. Supine and prone scenarios include single/twins cases and vice-versa. The bars compare the performance of three different algorithms: (1) conventional estimation using the Discrete Fourier Transform (DFT)^71–73^, representing the benchmark; (2) the proposed NLS estimation (NLS); and (3) the complete proposed solution (NLS+NMF), including the NMF-based RBM mitigation algorithm. The same preprocessing steps (phase demodulation and bandpass filtering) were used in all cases. The accuracy is being calculated as the percentage of time (in terms of valid processed windows) during which the final estimation from the radar is within a predefined error interval. For each case, the 3, 6 and 10 bpm accuracy were considered. For instance, a 6 bpm accuracy of 80% means that the magnitude of the error between radar estimation and the reference device was smaller than 6 bpm for 80% of the time.

It can be seen that the proposed techniques provided substantial improvement in all cases, with up to 17% of enhancement, and reaching a maximum 6 and 10 bpm accuracy of 79.3% and 93.1% respectively, in prone position. Figure 6e directly compares the performance of the complete proposed solution in each scenario, also including the average result of all processed sequences. An interesting outcome is related to the fact that the prone position produced on average the best results, even though the displacements verified from the back were smaller than the ones from the chest/abdominal region. This could be a result of the more uniform breathing motion of the posterior chest wall, since the ossification of the ribs begins at the back. Due to the higher flexibility of the anterior chest wall, abdominal and thoracic movements yield a more heterogeneous motion, thus making radar estimation more difficult. In addition, the prone position might lead to reduced body movements, thus reducing interference. Lastly, single and twin cases provided on average similar results, thus indicating that a second infant in the cot may not directly impact radar performance.

The root-mean squared error (RMSE) of each sequence was also calculated, and the average values for each scenario are presented in Fig. 6f, comparing the proposed techniques with the conventional DFT estimation. The RMSE is defined as

3 $$\begin{aligned} \text {RMSE} = \sqrt{\frac{1}{N} \sum _{n=0}^{N-1}{(B_n-\hat{B_n})^2}}, \end{aligned}$$

where $B_n$ and $\hat{B_n}$ represent, respectively, the reference and estimated breathing rates (in bpm), in the $n^{\mathrm{th}}$ processing window. It can be seen how the proposed solution outperforms the standard approach, providing significant and robust improvement over the different scenarios, and reducing the RMSE in all cases. The overall RMSE considering all valid measurements was 6.38 bpm. The measurements in prone position resulted in the best performance, with an RMSE close to 5 bpm. Supplementary File 4 shows the Bland-Altman analysis considering all scenarios and also comparing the standard DFT estimation and the complete proposed solution.

**Figure 7:**
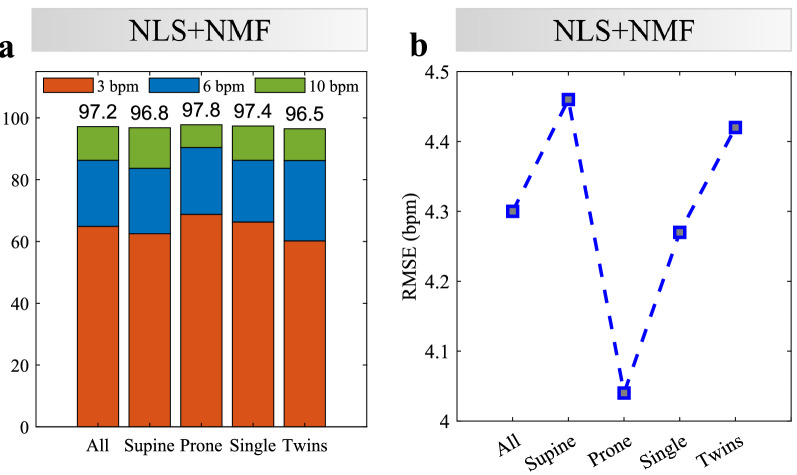
Average performance during minimal movement windows. (**a**) Accuracy for different scenarios. (**b**) Average RMSE for different scenarios.

The performance can also be analyzed considering only the moments without RBM or, at least, with reduced external interference. For identifying these moments we can use the RBM mitigation algorithm and look for the processing windows where no interfering components were captured by the time activation bases ${{\varvec{H}}}$. Given that spurious noise or additional frequency components may eventually be identified and filtered out as RBM, we accepted as “minimal movement windows” the ones in which a maximum of 2 bases were excluded by the algorithm. Figure 7 shows the obtained performance considering only these moments. The average 10 bpm accuracy for all measurements was higher than 97%, with the 6 bpm accuracy being higher than 80% in all scenarios. The average RMSE was 4.3 bpm, with the best result close to 4 bpm in prone position. In this case, all scenarios presented very similar performance, both in terms of accuracy and RMSE. The percentage of minimal movement windows in prone position was almost twice the number in supine position. This difference may confirm that the prone position leads to reduced random body movements and this is one of the reasons for the better results in this scenario.

This remaining RMSE is expected and can be explained if we take into consideration the premature conditions of the monitored infants (Supplementary Table 1), where the immaturity of their respiratory system and the diagnosed conditions may lead to irregular breathing patterns and thus hinder estimation. In addition, some transients may not be detected as RBM due to their specific characteristics, which may not agree with our assumption of strength and sparsity (for instance, hiccups which are periodic, or other continuous movements). Finally, some inaccuracy is also expected from the reference device, as these sensors (please refer to the Methods) suffer from imprecision and cardiac interference, specially in neonates with higher breathing rates and limited lung aeration^34,74^.

## Conclusions

The proposed radar-based solution was able to precisely recover the chest wall motion, allowing clear identification of different breathing patterns. This capability is the first step toward breathing frequency estimation and early non-invasive diagnosis of neonatal respiratory problems. In addition, most of the time the proposed algorithms provided reliable breathing frequency estimates, effectively reducing the effects of the RBM interference. The best performance was achieved when the infants were in prone position, with the 6 and 10 bpm accuracy reaching almost 80% and 93%, respectively. The overall RMSE was smaller than 7 bpm, with the best result close to 5 bpm in prone position. During minimal movements, the overall 10 bpm accuracy was higher than 97%, with the 6 bpm accuracy being higher than 80% in all scenarios. The average RMSE was 4.3 bpm, with the best result close to 4 bpm in prone position. These results can be interpreted as a proof-of-principle that the proposed radar-based approach has the potential for contactless breathing monitoring in the NICU. However, more experiments are underway to further reduce the vulnerability to artifacts, e.g. by using optimized algorithms of data processing and redundant technologies.

Finer radar calibration and a more precise setup adjustment would improve raw data quality and reduce ADC saturation. Further improvement of radar’s input data can still be achieved by shifting from CW to multiple-input multiple-output (MIMO) and frequency-modulated continuous-wave (FMCW) architectures. This change would allow not only range isolation from external interference, but also to steer the radar’s beam (field-of-view) directly to the monitored patient. The motion robustness can still be improved by tailoring the NMF algorithm according to the specific characteristics of the random body movement interfering signal. One particular case of interest would be the sparse NMF.

Finally, follow-up studies with more mature and healthy children of different ages would allow to identify pathologies, age-specific characteristics or confounding factors, and to adapt the experimental design accordingly. The best average results obtained at prone position point to the necessity of additional investigation on different clinical setups, where the radar could be also positioned under the mattress. Furthermore, with the aim to develop a complete contactless solution, an investigation of the radar capabilities for heart rate monitoring in this challenging environment is necessary, as well as moving to real-time processing.

## Methods

*Notation.* We are adopting the following notation: lower case boldface for vectors ${{\varvec{x}}}$, and upper case boldface for matrices ${{\varvec{X}}}$. The letter $\mathrm j$ represents the imaginary unit (i.e., ${\mathrm{j}} = \sqrt{-1})$, with the absolute value and angle operators given by the symbols $|(\cdot )|$ and $\angle (\cdot )$. The transpose, conjugate, and conjugate transpose operators are denoted respectively by the symbols $(\cdot )^{\mathrm{T}}$, $(\cdot )^*$, and $(\cdot )^{{\mathrm{H}}}$. The sets of *N*-dimensional vectors of complex and real numbers are represented by ${\mathbb {C}}^N$ and ${\mathbb {R}}^N$. The Euclidean norm of the vector ${{\varvec{x}}}$ is denoted by $||{{\varvec{x}}}||$. The Hadamard product is denoted by $\odot$.

*Ethics statement.* The study was designed in accordance to the Declaration of Helsinki, and approved by the regional ethics committee of Saarland (Saarbrücken, Germany), with reference number 276/17. Written informed consent was obtained from the parents prior to the data collection, and all the documentation and collected data were pseudonymized.

*Radar system.* The CW radar device used in this study is a prototype variant of the IEE’s VitaSense$^\text{\textregistered }$ sensor^75^, operating in the 24-GHz ISM band, with an illumination area of $76.5^{\circ }$ in azimuth, and $35.5^{\circ }$ in elevation. At the monitored distances, the low transmitted power leads to energy absorption rates 20 times below that of a cell phone^76^. The ADC sampling rate was 16 Hz, and the data acquisition was controlled by proprietary software on the external computer.

*Reference cabled device.* Throughout all measurements, the infants were connected to a reference monitor commonly employed in the NICU. In addition, respiration, heart rate and oxygen saturation signals were recorded using the VitaGuard$^\text{\textregistered }$ 3100 device^77^, cable-connected using 3 Kendall$^\text{\textregistered }$ neonatal 4203 electrodes^78^. This device measures the breathing movement through the electrodes attached to the infant’s chest (impedance pneumography), and provides the breathing rate with a resolution of 1 bpm, and an update rate of 1 s. The synchronization between reference and estimated frequency values was performed offline, based on the correlation between these signals. After processing an entire measurement (25 minutes), the resulting array with radar estimated frequency values is compared to the longer array with reference values, in a sliding window approach. The synchronization index was selected in order to maximize the correlation between these arrays. This procedure was performed automatically, using a Matlab routine.

*CW radar operational principle.* The transmitted CW signal can be written as

4 $$\begin{aligned} x_{\mathrm{T}}(t) = \sqrt{A_{\mathrm{T}}} \cos (2 \pi f_{\mathrm{c}} t + \phi (t)), \end{aligned}$$

where $A_{\mathrm{T}}$ and $f_{\mathrm{c}}$ are, respectively, the transmitted signal power and operating frequency, and $\phi (t)$ is the transmitter phase noise (local-oscillator). This signal is phase modulated by the target’s motion, and reflected to the radar for processing. The received signal from a target at nominal distance $d_0$ can be written as

5 $$\begin{aligned} x_{\mathrm{R}}(t) = \sqrt{A_{\mathrm{R}}} \cos \left( 2 \pi f_{\mathrm{c}} t - \frac{4\pi d_0}{\lambda } - \frac{4\pi d(t)}{\lambda }\right) , \end{aligned}$$

with $A_{\mathrm{R}}$ being the received power, $\lambda$ the operating wavelength, and *d*(*t*) representing the target motion. After demodulation and analog-to-digital conversion, and assuming correct I/Q imbalance compensation, the baseband I and Q signals can be represented as^79^

6 $$\begin{aligned} b_{\mathrm{I}}(t)= & {} \cos \left( \theta _0 + \frac{4\pi d(t)}{\lambda }\right) + B_{\mathrm{I}}, \end{aligned}$$

7 $$\begin{aligned} b_{\mathrm{Q}}(t)= & {} \sin \left( \theta _0 + \frac{4\pi d(t)}{\lambda }\right) + B_{\mathrm{Q}}, \end{aligned}$$

where $\theta _0=4\pi d_0/\lambda$ is the constant phase shift, and $B_{\mathrm{I}}$ and $B_{\mathrm{Q}}$ are DC offsets.

Under ideal conditions, the AD can be used for precise phase recovery. In this case, the displacement signal is reconstructed as^44^

8 $$\begin{aligned} x(t) = \frac{\lambda }{4 \pi } \cdot {\mathrm{unwrap}} \bigg (\arctan \bigg [ \frac{b_{\mathrm{Q}}(t)}{b_{\mathrm{I}}(t)} \bigg ] \bigg ) = \theta _0 + d(t), \end{aligned}$$

where the unwrap operation is necessary for removing possible phase discontinuities, caused by the restricted range of the arctangent function (wrapped phases around $(-\pi , \pi ]$ are expected when displacements are greater than $\lambda /4$). Before extracting the desired phase information, the DC components ($B_{\mathrm{I}}$ and $B_{\mathrm{Q}}$) must be compensated^73^. Given that the ideal chest wall (back-and-forth) movement describes an arc in the I/Q plane, this compensation is usually accomplished using an ellipse fitting algorithm. However, small displacements (small arcs), noise and/or external interference, usually compromise the fitting process, and lead to inaccurate DC compensation. In addition, the unwrap operation is also very sensitive to noise and interference, and may eventually accumulate errors, resulting in large distortions on the recovered displacement signal.

Using the CSD, the displacement signal can be reconstructed as^45^

9 $$\begin{aligned} x(t) = b_{\mathrm{I}}(t)+{\mathrm{j}} \cdot b_{\mathrm{Q}}(t) = {\overline{x}} + \exp \bigg \{ {\mathrm{j}} \left( \theta _0 + \frac{4 \pi d(t)}{\lambda } \right) \bigg \}, \end{aligned}$$

where ${\overline{x}} = B_{\mathrm{I}} + {\mathrm{j}} B_{\mathrm{Q}}$ represents the combined DC offset. However, in this case, this DC term does not affect the relevant components of the recovered signal, and, in practice, it can be easily extracted by subtracting the average of the time-domain processing window. Despite additional higher order harmonics, for small displacements (in relation to the operating wavelength), the recovered signal *x*(*t*) approximates the true chest wall movement *d*(*t*), and the relevant frequency content is preserved. Therefore, besides being more robust to the DC offsets and external interference, the CSD simplifies the demodulation procedure. A detailed description of AD and CSD methods can be found in^44^ and^80^.

*Nonnegative matrix factorization.* The magnitude spectrogram $|{{\varvec{X}}}| \in {\mathbb {R}}^{F\times T}$ of the recovered displacement signal is obtained through the STFT of *x*(*t*), where *F* and *T* are, respectively, the number of frequency and time bins used in the STFT operation. Given that we aim to reconstruct the time version of the RBM-filtered signal, the STFT weighting window must comply with the constant overlap-add property^81^.

The NMF is thus applied to the magnitude of ${{\varvec{X}}}$, and the factorization can be achieved through an optimization problem given by

10 $$\begin{aligned}&\mathop {\mathrm{minimize}}\limits _{{{\varvec{W}}},\,{{\varvec{H}}}} {\mathcal {L}}\left( {{\varvec{X}}},\,{{\varvec{W}}}{{\varvec{H}}}\right) \nonumber \\&{\mathrm{subject\,to}} \, {{\varvec{W}}}\succeq 0,\, {{\varvec{H}}}\succeq 0, \end{aligned}$$

with ${{\varvec{H}}}\in {\mathbb {R}}^{K\times T}$ and ${{\varvec{W}}}\in {\mathbb {R}}^{F\times K}$. The symbol “$\succeq$” denotes entry-wise non-negativeness and ${\mathcal {L}}(\cdot ,\cdot )$ represents a generic similarity metric between $|{{\varvec{X}}}|$ and ${{\varvec{W}}}{{\varvec{H}}}$. The Euclidean (Frobenius) distance is commonly used and, in doing so, simple gradient descent can be used for minimizing the objective function^58^. The predefined number of basis components *K* should be selected considering the different frequency components that may be present in the calculated spectrogram, including the breathing frequency and eventual RBM interference. For the long-term monitoring, we have empirically selected $K=11$.

The identification of the time activation basis corresponding to the RBM is based on an adaptive amplitude threshold. The breathing pattern is affected by several factors (subject, gender, age, health condition, posture) and efficient interference detection requires an adaptive strategy. The RBM interference can be characterized by its distinct behavior which contrasts with the constant and periodic nature of breathing. Therefore, subsequent to NMF decomposition, we are looking for strong (stronger than average) and sparse (short duration in relation to the processing window) time activation basis ${{\varvec{h}}}_i$ in ${{\varvec{H}}}$. This can be accomplished simply by comparing the local energy in each time component of ${{\varvec{h}}}_i$, with the average energy in ${{\varvec{H}}}$ for the current processing window (under regular conditions, that would correspond to the average breathing energy). This average energy acts as the threshold and, by the nature of its computation, it changes for each window and reflects the signal strength therein. Therefore, the threshold will be automatically adjusted for each processing window accordingly. Additionally, the sparsity is verified by checking if the remaining components of the selected basis have negligible amplitude.

The magnitude of the RBM-filtered spectrogram can be reconstructed as

11 $$\begin{aligned} |{\hat{{{\varvec{X}}}}}| = \sum _{i=1}^{K} s_i {{\varvec{w}}}_i {{\varvec{h}}}^{\mathrm{T}}_i, \end{aligned}$$

where $s_i$ indicates whether the $i^{\mathrm{th}}$ basis corresponds to RBM or not, i.e. $s_i = 0$ when RBM is present in basis ${{\varvec{h}}}_i$, or $s_i = 1$ otherwise. In order to synthesize the time-domain filtered signal ${\hat{x}}(t)$, it is first necessary to obtain the phase of the filtered spectrogram. A common practice is to use a Wiener-like filtering approach, which translates into reusing the phase of the original mixed spectrogram ${{\varvec{X}}}$^82^. The inverse STFT is finally applied to ${\hat{{{\varvec{X}}}}} = |{\hat{{{\varvec{X}}}}}| \odot \angle {{{\varvec{X}}}}$, replicating the same window configuration (duration, weights and overlap) as in the initial STFT. In this work we are using standard rectangular windows with 3 s of duration, 2 s of overlap and zero-padding to 256 samples.

The RBM method is applied to every processing window. The method is capable of automatically detecting and removing the RBM interference, based on the adaptive amplitude threshold and the sparsity check. No manual annotation is needed. In the absence of RBM or other interfering effects, the recovered signal will contain only the constant and periodic chest wall movement, and the resulting time activation bases ${{\varvec{H}}}$ will reflect that. In this case, the adaptive amplitude threshold will not identify any candidate basis containing RBM, and the filtered signal will be approximately the same as in the input of the RBM block.

*NLS estimation.* The breathing displacement signal *d*(*t*) can be modeled as a sum of $L_k$ harmonically related complex sinusoids, having frequencies that are integer multiples of a fundamental frequency $\omega _k>0$. After sampling for $n \in \{0, \cdots ,N-1\}$, such a signal can be written as^65^

12 $$\begin{aligned} d(n) = \sum _{k=1}^{K} d_k(n) = \sum _{k=1}^{K} \sum _{l=1}^{L_k} a_{k,l} {\mathrm{e}}^{{\mathrm{j}} \omega _k l n}, \end{aligned}$$

where $a_{k,l} = A_{k,l} {\mathrm{e}}^{{\mathrm{j}} \phi _{k,l}}$ is the complex amplitude of the *l*th harmonic, $L_k$ represents the number of harmonics (the model order), and *K* refers to the number of components (point scatters).

The NLS estimates are obtained by minimizing the Euclidean norm of the difference between the recovered and filtered displacement signal ${\hat{d}}(t)$, and the displacement signal model in (12). First, let us consider a single source *k*, and define ${{{\varvec{d}}}_k = [\; d_k(0) \; \cdots \; d_k(N-1) \;]^{\mathrm{T}} \in {\mathbb {C}}^N}$, the vector consisting of *N* consecutive samples of $d_k(n)$, which can be expressed as

13 $$\begin{aligned} {{\varvec{d}}}_k = {{\varvec{Z}}}_k {{\varvec{a}}}_k, \end{aligned}$$

with ${{\varvec{a}}}_k = [\; A_{k,1} {\mathrm{e}}^{{\mathrm{j}} \phi _{k,1}} \; ... \; A_{k,L_k} {\mathrm{e}}^{{\mathrm{j}} \phi _{k,L_k}} \;]^{\mathrm{T}}$ being the vector containing the complex amplitudes of the harmonics, and the matrix ${{{\varvec{Z}}}_k \in {\mathbb {C}}^{N \times L_k}}$ having a Vandermonde structure, being constructed from the $L_k$ complex sinusoidal vectors as

14 $$\begin{aligned} {{\varvec{Z}}}_k = [\; {{\varvec{z}}}(\omega _k) \; {{\varvec{z}}}(2 \omega _k) \; ... \; {{\varvec{z}}}(L_k \omega _k) \;], \end{aligned}$$

with ${{\varvec{z}}}(\omega ) = [\; 1 \; {\mathrm{e}}^{{\mathrm{j}}\omega } \; ... \; {\mathrm{e}}^{{\mathrm{j}}\omega (N-1)} \;]^{\mathrm{T}}$. Writing ${\hat{d}}(t)$ as the vector ${{\hat{{{\varvec{d}}}}} = [\; {\hat{d}}(0) \; \cdots \; {\hat{d}}(N-1) \;]^{\mathrm{T}} \in {\mathbb {C}}^N}$, the NLS estimates ${\hat{\omega }}_k$ and $\hat{{\varvec{a}}}_k$ are finally obtained by solving the problem^69^

15 $$\begin{aligned}&\mathop {\mathrm{minimize}}\limits _{\omega _k,{{\varvec{a}}}_k} || {\hat{{{\varvec{d}}}}}-{{\varvec{Z}}}_k {{\varvec{a}}}_k ||^2. \end{aligned}$$

By first minimizing (15) with respect to the complex amplitudes ${{\varvec{a}}}_k$ we obtain the estimate ${\hat{{{\varvec{a}}}}}_k = ({{\varvec{Z}}}_k^{\mathrm{H}}{{\varvec{Z}}}_k)^{-1}{{\varvec{Z}}}_k^{{\mathrm{H}}}{\hat{{{\varvec{d}}}}}$, which when plugged into (15) leads to^69^

16 $$\begin{aligned} {\hat{\omega }}_k = \arg \max _{\omega _k} {\hat{{{\varvec{d}}}}}^{{\mathrm{H}}} {{\varvec{Z}}}_k ({{\varvec{Z}}}_k^{{\mathrm{H}}} {{\varvec{Z}}}_k)^{-1} {{\varvec{Z}}}_k^{{\mathrm{H}}} {\hat{{{\varvec{d}}}}}. \end{aligned}$$

Assuming $N \gg 1$, then ${{\varvec{Z}}}_k^{{\mathrm{H}}} {{\varvec{Z}}}_k \approx N\cdot {{\varvec{I}}}_{L_k}$. Thus, considering only a single dominant breathing scatter (i.e. $K=1$, so that we can drop the index *k*), we have

17 $$\begin{aligned} {\hat{\omega }} \approx \arg \max _{\omega } ||{{\varvec{Z}}}^{{\mathrm{H}}} {\hat{{{\varvec{d}}}}} ||^2. \end{aligned}$$

The matrix product ${{\varvec{Z}}}^{{\mathrm{H}}} {\hat{{{\varvec{d}}}}}$ can be efficiently implemented using an FFT algorithm and a linear grid search over the candidate frequencies $\left\{ 0, \frac{2\pi }{N},\cdots ,\frac{2\pi }{N}(N-1)\right\}$. The estimator thus reduces to a summation of the breathing harmonics over the power spectral density of the recovered displacement signal ${\hat{d}}(t)$. Usually, a few harmonics may be present in ${\hat{d}}(t)$. However, the small motion amplitudes of the monitored patients imply a reduced SNR, where higher-order harmonics will often be masked by noise. Therefore, in this work, we have adopted $L_k=2$. Additionally, the search interval was limited to $\pm 5$ bpm around the initial time domain estimation.

## Supplementary Information

Supplementary Information.

## Data Availability

The original dataset analyzed during the current study is available for download at https://radarmimo.com/.

## Code availability

Matlab reading functions for both radar and reference device are available for download at https://radarmimo.com/. Please refer to the Supplementary File 1 for a brief description of the codes.
