# Supplementary material for: Contactless radar-based breathing monitoring of premature infants in the neonatal intensive care unit
Source: Sci Rep. 2022 Mar 25;12:5150. doi: 10.1038/s41598-022-08836-3 (PMC8956695; doi:10.1038/s41598-022-08836-3)
Supplement: Supplementary file 1 — Supplementary Information. [file 41598_2022_8836_MOESM1_ESM.pdf]

Nature Scientific Reports

“Contactless radar-based breathing monitoring of premature infants in the neonatal intensive care unit”

Gabriel Beltrão, Regine Stutz, Franziska Hornberger, Wallace A. Martins, Dimitri Tatarinov, Mohammad Alaee-Kerahroodi, Ulrike Lindner, Lilly Stock, Elisabeth Kaiser, Sybelle Goedicke-Fritz, Udo Schroeder, Bhavani Shankar M.R., Michael Zemlin

Supplementary File 1: Matlab codes

- readRadarRaw.m: simple function for reading radar .csv files with the raw data in I and Q format.
- testReadRadarRaw.m: procedure for test the reading of radar files and plot.

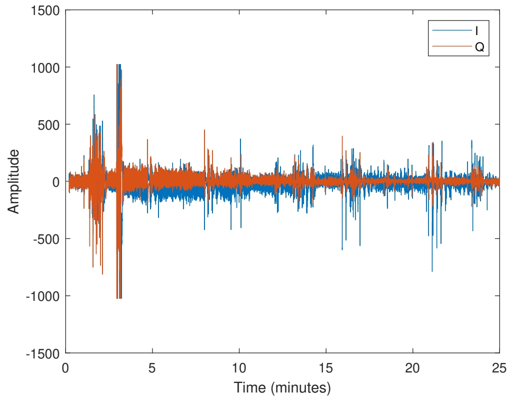

Fig. 1: **Radar raw data in I and Q format.**

- readReferenceData.m: simple function for reading the reference device .edf files, with the raw data and reference frequency values.
- testReadReferenceData.m: procedure for test the reading of reference files and plot.

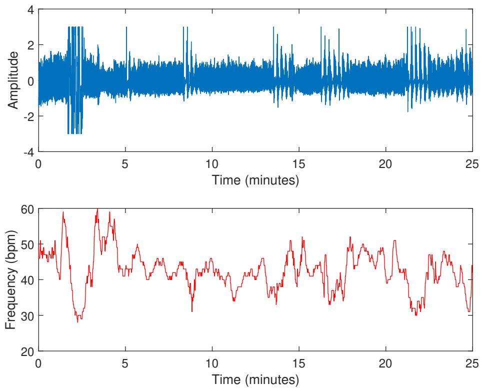

Fig. 2: **Reference cabled device raw data (top) and correspondent frequency values (bottom).**

Supplementary File 2: Patient's information

- Supplementary Table 1: Summary of patient's information.

patient	01	02	03	04	05	06	07	08	09	10	11	12
sex	m	f	m	m	m	m	m	f	m	f	m	m
gestation age [weeks]	28	28	36	32	28	28	34	34	35	26	27	30
gestation weight [g]	1495	1250	2080	1990	850	760	2430	2210	1490	990	1145	1940
gestation length [cm]	40	38	45	46	36	35	48	48	37	34	38	41
age at measurement [days]	63 ± 1	63 ± 1	10 ± 1	9 ± 2	91 ± 1	91 ± 1	14 ± 1	14 ± 1	18 ± 2	50 ± 2	52 ± 1	28 ± 1
body weight at measurement [g]	2650	2618	2165	2165	2165	2165	2165	2165	2165	2165	2165	2165
body length at measurement [cm]	47	47	46	46	-	-	50	50	45	-	45	50
measurements per patient	3	3	3	3	3	3	3	3	3	3	3	3
time per measurement [min]	25	25	25	25	25	25	25	25	25	25	25	25
diagnosis												
respiratory distress syndrome	x	x	-	-	x	x	x	-	-	x	x	-
apnea bradycardia syndrome	x	x	-	x	x	x	x	x	x	x	x	x
bronchopulmonary dysplasia	-	-	-	-	-	-	-	-	-	x	x	-
respiratory adaptive disorder	-	-	-	-	-	-	-	x	x	-	-	x

- Supplementary Fig. 1: Radar distance in each measurement.

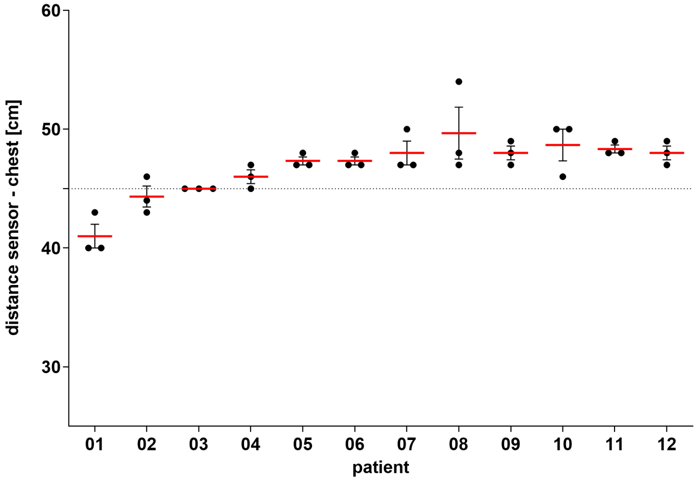

Fig. 1: **Radar distance in each measurement.**

Supplementary File 3: Patient's protocols

- Supplementary Table 2a: patient's protocols (1-3). [1]

measure-ment	co-bedding	position during measurement			lid	interventions	transients
		prone	supine	side			
1.1	yes		x		half-opened	12:34-13:25: nurse at sibling<br>17:39-18:33: mother at sibling<br>21:30-22:01: mother at patient	03:17-09:07: hiccups<br>24:02-25:00: crying
1.2	yes	x			half-opened	06:26-06:49 nurse at patient	04:05-04:26: unsettled<br>07:00-08:00: unsettled<br>11:39-12:00: grunting<br>14:35-15:13: movement<br>17:57-18:15: movement
1.3	yes		x		half-opened	18:17-19:00: mother at patient	09:12-10:20: movement / yawn<br>11:50-12:35: movement<br>14:50-16:30: movement<br>22:20-24:42: grunting/movement
2.1	yes		x		half-opened	20:00-20:35: nurse at bed	02:48-03:20 sibling unsettled<br>06:15-07:40 unsettled<br>08:28-10:50 movement/grunting<br>11:39-12:00 sibling movement<br>14:00-14:45: grunting / movement<br>15:30-15:35: grunting<br>17:00-18:00: sibling crying<br>24:00-25:00: yawn
2.2	yes		x		half-opened	16:25-25:00: nurses present in room	02:20-03:25: movement full body<br>03:25-05:47: movement hand<br>06:23-07:50: movement / yawn<br>09:28: pulls cables<br>10:07-11:50: movement<br>13:31-14:29: movement hand<br>18:12-22:35: movement hand
2.3	yes		x		half-opened	05:06-05:30: mother at patient	03:14-04:00: unsettled<br>07:00-07:30: movement / yawn<br>09:00-10:00: unsettled<br>10:48-13:00: unsettled<br>14:00-16:30: unsettled<br>18:17-19:05: unsettled<br>23:04-23:58: unsettled<br>24:28-25:00: yawn
3.1	no	x			closed	04:40-05:10: nurse at bed	07:00-08:00: movement<br>10:10-11:05: movement/yawn<br>16:54-17:26: unsettled<br>18:38-19:00: movement hand<br>19:08-19:30: movement/yawn<br>21:30-22:07: movement/yawn
3.2	yes	x			closed	-	03:50-04:05: sibling movement<br>08:30-09:15: movement/yawn<br>09:40-10:40: sibling movement<br>11:06-11:28: movement/yawn<br>12:18-12:58: sibling movement<br>18:13-18:33: movement<br>19:46-20:07: movement/grunting<br>21:48-22:05: movement
3.3	yes			x	closed	-	02:47-03:13: movement hand<br>04:03-04:29: sibling movement<br>06:47-07:26: unsettled<br>09:44-10:47: sibling movement<br>11:07-11:26: movement/yawn<br>13:24-16:27: sibling movement<br>20:07-20:39: sibling movement<br>21:47-22:23: movement<br>22:45-25:00: sibling movement

[1]The timestamps indicate the moments they happened, starting from the beginning of the measurement (00:00" to 25:00").

- Supplementary Table 2b: patient's protocols (4-6). [1]

measure-ment	co-bedding	position during measurement			lid	interventions	transients
		prone	supine	side			
4.1	no		x		closed		03:00-03:10: movement/yawn<br>15:10-15:40: unsettled<br>16:32-16:42: twitch<br>18:52-19:00: movement<br>19:43-21:27: movement/grunting<br>23:28-23:57: movement<br>24:04-25:00: twitch
4.2	no		x		closed	02:54: nurse walks along the bed<br>06:52: nurse walks along the bed<br>07:39-08:13: nurse at the bed<br>09:31: nurse walks along the bed<br>13:10: nurse walks along the bed<br>15:50: nurse walks along the bed<br>17:28: nurse walks along the bed<br>20:40: nurse at the bed	03:27-05:03: movement<br>06:00-06:30: hands movement
4.3	no	x			closed	01:07: nurse walks along the bed<br>05:13: nurse walks along the bed<br>06:03: nurse walks along the bed	03:36: grunting<br>05:35-05:50: yawn / movement<br>07:34-07:58: movement<br>09:00-09:30: yawn/movement<br>09:37-11:43: unsettled<br>12:21-13:20: movement hand<br>13:20-14:10: movement<br>15:20-15:40: movement head<br>16:25-19:00: movement hand<br>21:00-24:24: movement
5.1	no		x		closed	20:47-25:00: four nurses present in the room	04:23-06:28: grunting / movement<br>08:57-11:42: unsettled<br>13:12-13:36: unsettled<br>13:55-15:42: movement<br>19:36-20:25: yawn / movement<br>24:19-24:29: movement
5.2	no		x		closed	-	00:16-03:54: crying<br>04:06-04:35: crying<br>09:26-11:46: movement/crying<br>15:07-16:26: movement<br>23:53-24:15: movement
5.3	no	x			closed	-	02:20-04:45: unsetted / grunting<br>06:58-07:30: grunting / movement<br>10:20-12:13: unsettled / grunting<br>14:17-14:50: grunting<br>15:59-16:20: unsettled / movement<br>18:33-19:12: grunting/movement<br>23:35-24:06: grunting/movement
6.1	no		x		closed	12:43-13:22: nurse moves bed<br>16:58-17:23: nurse at the bed	02:56-04:28: movement/grunting<br>05:05-05:20: movement<br>06:31-07:40: grunting / yawn<br>08:17-10:00: movement<br>12:25-15:55: movement<br>18:12-18:58: grunting/movement<br>21:09-22:25: movement<br>23:30-24:33: grunting<br>24:23-25:00 crying
6.2	no	x			closed		05:52-06:35: unsettled<br>11:13-12:10: grunting / movement<br>14:10-14:50: grunting / movement<br>16:42-17:14: grunting<br>18:59-19:43: unsettled<br>22:50-23:28: unsettled
6.3	no		x		closed		04:22-05:36: grunting / movement<br>08:39-08:57: yawn<br>09:04-09:43: grunting / movement hands<br>10:32-11:30: movement<br>11:45-12:16: unsettled<br>14:11-16:03: grunting / movement<br>18:11-19:21: movement<br>21:07-22:23: movement

[1] The timestamps indicate the moments they happened, starting from the beginning of the measurement (00:00" to 25:00").

- Supplementary Table 2c: patient's protocols (7-9). [1]

measure-ment	co-bedding	position during measurement			lid	interventions	transients
		prone	supine	side			
7.1	no		x		closed	-	05:10-05:35: unsettled<br>11:06-12:45: movement<br>17:04-17:34: unsettled
7.2	yes		x		closed	-	02:07-02:29: unsettled<br>02:44-03:54: sibling unsettled<br>08:19-10:01: yawn / movement<br>11:22-11:47: sibling unsettled<br>14:18-14:45: sibling unsettled<br>15:09-15:17: movement<br>16:27-16:42: unsettled<br>18:55-19:08: sibling unsettled<br>19:50-20:18: unsettled
7.3	yes	x			closed	02:50-03:15: fasten O2-Sensor	01:38-02:14: unsettled
8.1	no		x		closed	-	all time hands movement<br>05:41-06:39: movement<br>08:19-09:01: yawn / movement<br>13:02-13:17: yawn / movement<br>16:00-16:48: yawn / movement<br>17:38-18:00: movement<br>19:21-20:21: yawn / movement<br>22:18-22:35: yawn / movement
8.2	yes		x		closed	-	02:53-03:33: grunting / movement<br>05:14-06:04: grunting / movement<br>12:18-13:08: grunting / movement<br>16:15-17:02: movement<br>19:15-20:17: unsettled / movement
8.3	yes	x			closed	06:00-06:41: nurse at sibling	02:34-02:53: grunting / movement<br>06:20-06:47: unsettled<br>10:34-11:08: grunting
9.1	no		x		closed	-	11:12-11:28: yawn<br>12:56-13:11: grunting<br>15:13-15:42: unsettled / movement<br>19:32-19:56: grunting<br>20:00-20:12: movement
9.2	no		x		closed	-	01:05-02:38: movement / grunting<br>04:11-04:18: grunting<br>04:58-06:49: movement<br>11:00-11:11: movement<br>12:04-12:51: movement<br>15:07-15:37: movement<br>19:49-20:51: movement<br>22:13-22:27: grunting<br>23:40-25:00: movement / grunting
9.3	no		x		closed	-	01:00-01:59: grunting / movement<br>03:49-03:57: grunting / movement<br>08:55-09:38: grunting / movement<br>10:27-10:59: grunting / movement<br>15:01-16:03: grunting / movement<br>17:45-18:02: grunting / movement<br>18:52-19:11: grunting / movement<br>23:23-24:19: grunting / movement

[1]The timestamps indicate the moments they happened, starting from the beginning of the measurement (00:00" to 25:00").

- Supplementary Table 2d: patient's protocols (10-12). [1]

measure-ment	co-bedding	position during measurement			lid	interventions	transients
		prone	supine	side			
10.1	no		x		closed	-	01:13-01:37: movement<br>03:24-04:25: yawn / grunting / movement<br>06:43-07:37: movement / grunting<br>10:58-11:28: grunting / movement<br>15:18-16:27: yawn / movement<br>18:30-19:27: movement
10.2	no	x			closed	-	05:49-06:37: grunting / movement<br>09:10-09:23: movement<br>11:07-11:46: movement<br>12:45-13:09: movement / grunting<br>14:33-15:13: grunting<br>20:04-20:10: yawn / movement<br>22:58-23:04: yawn
10.3	no	x			closed	-	04:01-05:13: movement<br>09:28-09:45: movement / grunting<br>13:18-13:56: grunting / movement<br>17:17-18:14: grunting / movement<br>21:07-22:14: grunting / movement
11.1	no		x		closed	-	02:04-03:21: grunting / movement<br>05:16-05:33: yawn / movement<br>09:29-10:05: yawn / movement<br>12:47-14:20: grunting / yawn / movement<br>18:31-18:46: grunting / movement<br>18:47-19:29: grunting<br>20:59-21:33: movement<br>23:52-24:23: grunting / movement
11.2	no		x		closed		02:24-03:46: yawn / movement<br>06:28-06:37: grunting<br>07:11-07:56: movement<br>09:13-09:41: movement<br>11:27-11:52: yawn / movement<br>15:24-16:58: movement / grunting<br>21:39-22:31: movement / grunting
11.3	no		x		closed	09:48-10:35: nurse walks along the bed	02:30-02:52: movement<br>05:55-06:12: grunting / movement<br>20:47-21:05: grunting / movement<br>23:15-24:31: movement
12.1	no	x			closed	05:00: nurse walks along the bed<br>06:22: nurse walks along the bed	02:36-03:29: movement / yawn<br>09:02-09:51: movement / grunting<br>09:58-19:44: hiccups<br>11:21-12:10: movement<br>15:50-16:45: yawn / movement<br>17:24-17:37: movement<br>18:35-19:29: movement<br>20:15-20:43: movement<br>22:00-22:26: yawn / movement
12.2	no	x			closed	-	01:38-02:26: grunting<br>07:32-08:02: grunting / movement<br>09:34-09:40: movement<br>10:37-11:05: yawn / movement<br>15:33-16:01: grunting<br>17:03-17:16: grunting<br>18:43-18:53: yawn<br>20:30-20:42: grunting<br>23:36-25:00: hiccups
12.3	no		x		closed	01:12-01:17: mother walks along the bed<br>10:47-11:16: nurse at bed	01:21-02:31: movement<br>04:55-05:02: yawn / movement<br>07:46-09:02: movement / yawn<br>09:15-09:24: movement<br>10:40-11:18: movement<br>13:08-13:37: movement<br>15:33-16:04: movement / grunting<br>16:11-16:53: yawn / movement<br>16:58-17:24: crying<br>19:07-20:01: movement

[1]The timestamps indicate the moments they happened, starting from the beginning of the measurement (00:00" to 25:00").

Supplementary File 4: Bland-Altman's analysis

Figure 1a shows the Bland-Altman plot considering all measurements for the complete solution. The mean bias was 0.262, with 95% upper and lower limits of agreement (LOAs) of 12.01 bpm and -11.48 bpm, respectively. Figure 1b shows the Bland-Altman plot considering all valid estimates for a single measurement (7.3), in prone position (please refer to the Supplementary File 3). It also compares the performance using standard DFT estimation and the complete proposed solution. The mean bias was -0.296, with LOAs of 7.64 bpm and -8.24 bpm, respectively.

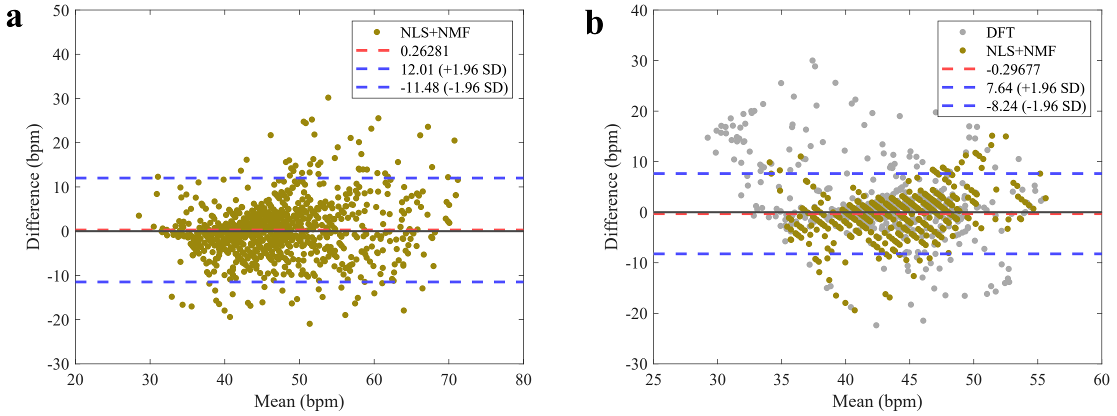

Fig. 1: **Bland-Altman analysis. a,** All measurements for the complete solution. **b,** Single measurement, comparing the standard DFT estimation and the proposed solution.
